# Usefulness of cell-free and concentrated pleural effusion reinfusion therapy in terminal cancer patients

**DOI:** 10.20407/fmj.2024-034

**Published:** 2025-11-05

**Authors:** Hiroyuki Fujisaki, Masanobu Usui, Akihiro Ito, Miyo Murai, Norimasa Tsuzuki, Akihiko Futamura, Kazuki Imai, Yoshinori Itani

**Affiliations:** 1 Department of Surgery and Palliative Medicine, Fujita Health University, School of Medicine, Toyoake, Aichi, Japan; 2 Department of Medical Technology, Clinical Examination Division, Fujita Health University Nanakuri Memorial Hospital, Tsu, Mie, Japan

**Keywords:** Malignant pleural effusion, Cell-free and concentrated pleural effusion reinfusion therapy (CPRT), Cell-free and concentrated ascites reinfusion therapy (CART), Nutritional status, Clinical manifestations

## Abstract

**Objective:**

Patients with advanced or recurrent cancer often suffer from excessive fluid retention, which substantially affects their quality of life. There are no detailed reports regarding cell-free and concentrated pleural effusion reinfusion therapy (CPRT). Thus, we investigated the usefulness of CPRT for pleural effusions in terminal cancer patients.

**Methods:**

We evaluated the efficacy and complications of CPRT in 29 patients treated at Fujita Health University Nanakuri Memorial Hospital between April 2016 and August 2020. Nutritional status before and after CPRT was evaluated by measuring serum albumin (Alb) and transthyretin (TTR) levels as indicators. Clinical symptoms were assessed to evaluate the following nine items: pain, general fatigue, anorexia, dyspnea, depression, nausea, insomnia, constipation, and dry mouth.

**Results:**

CPRT was performed a total of 71 times, with a median of one session per patient. Median volume of drained pleural fluid was 800 mL, and the median volume of reinfused fluid was 120 mL. Regarding nutritional status before and after CPRT, serum Alb and TTR values did not decrease significantly and were maintained. Clinical symptoms showed a tendency toward improvement; dyspnea, depression, and insomnia improved significantly.

No correlation was observed between the volume of pleural fluid drainage and the reduction of dyspnea, but there was a mild correlation with general fatigue and nausea. Conversely, constipation was mildly inversely correlated with the volume of pleural fluid drainage.

**Conclusion:**

CPRT for pleural effusions in patients with terminal cancer was suggested to maintain nutritional status and may be useful in improving clinical symptom.

## Introduction

Patients with advanced or recurrent cancer often suffer from excess fluid retention, including anasarca, pleural effusion, and ascites, along with disease progression and cachexia development. Pleural effusion may lead to respiratory distress and necessitate the administration of oxygen and opioids, thereby contributing to a decline in ADLs. Therefore, controlling symptoms caused by pleural effusions significantly impacts the patient’s quality of life (QOL). The first step in treatment is to administer diuretics and reduce the volume of infused fluid. However, if a large pleural effusion accumulates, especially if breathing difficulties are present, drainage is often performed. Currently in Japan, in addition to drainage, cell-free and concentrated ascites reinfusion therapy (CART) is widely used as a treatment for massive ascites associated with peritoneal metastases of cancer. CART is a therapeutic procedure in which ascitic fluid is processed using a filtration device to remove bacteria, cancer cells, and blood cell components. Protein components, including albumin and globulin, are then collected and reinfused into the same patient. Reinjection of recovered proteins helps maintain plasma colloid osmotic pressure, suppresses reaccumulation, enhances the patient’s general and nutritional status, thereby improving QOL, and avoids infectious and immunological side effects associated with plasma-derived albumin products.^1^ Conversely, detailed reports on CPRT for pleural effusion are limited. In this study, we report the effects of CPRT on clinical symptoms and nutritional indices in patients with advanced or recurrent cancer.

## Methods

Among the 1,328 patients admitted for palliative care to Fujita Medical University Nanakuri Memorial Hospital over the 4 years and 5 months from April 2016 to August 2020, 29 (2.2%) underwent CPRT.

This is retrospective study. At the time of admission, all patients were informed that test results and imaging data obtained during hospitalization might be used for research purposes with appropriate consideration for privacy, and written informed consent was obtained; no patients declined to provide consent for participation in this study. For patients in whom CPRT was indicated, the potential benefits and risks, including possible complications, were thoroughly explained to the patients and their families, and CPRT was performed only after written informed consent was obtained. CPRT was indicated for patients with pleural effusion that did not improve despite treatment with diuretics and fluid restriction, specifically those presenting with dyspnea. However, as with CART, CPRT was not indicated for cases where infection was strongly suspected based on, fever of ≥38°C, and elevated CRP levels, or for cases with a serum total bilirubin level of ≥5 mg/dL, or for cases with marked hemolysis in bloody pleural effusion.^1^ Furthermore, some patients are admitted for palliative care and do not wish to undergo pleurodesis or CPRT. In such cases, the patient’s and family’s wishes were prioritized. Pleurocentesis was not performed, and efforts were made to control symptoms by administering opioids and other medications.

This study was approved by the Ethics Review Committee of Fujita Health University (HM16-401). The number of drainages performed, the volume of drained pleural fluid, the presence or absence of complications, and changes in clinical symptoms were examined as endpoints. To evaluate nutritional status before and after CPRT, serum albumin (Alb: g/dL) and transthyretin (TTR: mg/dL) levels were compared in 11 patients whose blood biochemical tests were measured 3 weeks before and 3 weeks after CPRT. Blood samples were collected before breakfast during fasting and analyzed using the same serum. TTR values were measured using the TIA method using Espa TTR II reagent, and Alb values were measured using the modified BCP method using the AU reagent ALB. These blood samples were analyzed using the AU680 Automatic analyzer (BECKMAN COULTER, Osaka, Japan). The reference ranges for serum TTR and Alb concentrations were 22–44 mg/dL and 4.0–5.0 g/dL, respectively.

**Figure 1:**
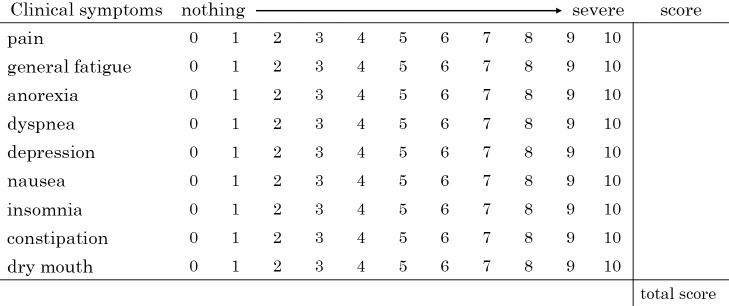
Overall assessment score

Clinical symptoms were evaluated by scoring the following nine items on an 11-point scale from 0 to 10, using a numerical rating scale^2^: Pain, general fatigue, anorexia, dyspnia, depression, nausea, insomnia, constipation, and dry mouth. The scores for these nine items were then summed to create an overall clinical symptom score (Figure 1). The patient is interviewed and assessed upon admission and subsequently once a week.

Pleural drainage was performed under local anesthesia using an aspiration kit (COVIDIEN, Argyle 8Fr). The collected fluid was stored in a specialized collection bag (Asahi Kasei Medical). The collected fluid was filtered and concentrated using the AHF-MO ascites filter and AHF-UF ascites concentrator (Asahi Kasei Medical), and reinfused at a rate of approximately 100 mL/h. In other words, after initially separating and removing cancer cells, blood cells, bacteria, and fibrin from the original pleural fluid using a filtration membrane, excess water and electrolytes were removed with a concentration membrane to produce an albumin-globulin concentrate with a final total volume approximately one-tenth of the original, which was then reinfused. As rapid drainage of a large volume of pleural fluid may lead to re-expansion pulmonary edema, we adhered to the recommendations of the Japanese Society for Palliative Medicine’s “Guidelines for the Palliation of Dyspnea in Patients with Advanced Disease” and limited each drainage session to approximately 1000 to 1500 mL.^3^ In principle, premedication with steroids for purposes such as fever prevention is not administered.

### Statistical test

Continuous variables were analyzed using the Mann–Whitney U test, and categorical variables were analyzed using Fisher’s exact test. A p-value of less than 0.05 was considered statistically significant. Spearman’s rank correlation coefficient (r) was calculated, and a p-value of less than 0.05 was considered statistically significant. 0.4≤r≤1.0 indicated a positive correlation, and –1.0≤r≤–0.4 indicated a negative correlation.

## Results

As background factors, the median age was 74 years (range: 49–89 years), with a male-to-female ratio of 14:15. The primary lesions mainly included lung cancer in 12 cases (40.0%), bile duct cancer and breast cancer in three cases each (10.0%), followed by colon cancer and kidney/ureteral cancer in two cases each, and stomach, pancreatic, peritoneal, uterine, thymus, mandibular cancer, and intrathoracic osteosarcoma in one case each.

Eight cases were associated with metastatic lung cancer, including carcinomatous pleurisy (Table 1).

CPRT was performed 71 times in total, with a median of one time (range: 1–14 times). More than half of the cases were performed only once, with a maximum of 14 times. Median volume of drained pleural fluid was 800 mL (range: 200–2000 mL), and the median volume of reinfused fluid was 120 mL (range: 50–330 mL) (Table 2). Complications included fever ≥38°C in six patients (8.4%) and pneumothorax requiring decompression in one patient (1.4%).

**Figure 2:**
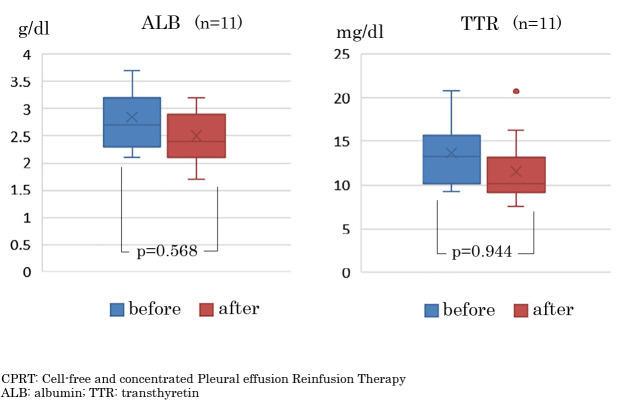
Changes in serum albumin and transthyretin levels before and after CPRT

In 11 cases, nutritional status changes could be assessed, as blood biochemistry tests were performed within 3 weeks before and after CPRT. Serum Alb levels were maintained with no significant difference, with a median of 2.4 (1.4–3.7) g/dL after CPRT compared to a median (interquartile range) of 2.3 (1.9–3.7) g/dL before CPRT (p=0.568). Serum TTR showed no significant difference after CPRT, with a median of 10.2 (7.6–20.7) g/dL, compared to a median of 13.3 (9.3–20.8) g/dL before CPRT (p=0.944) (Figure 2).

Cumulative evaluation of clinical symptoms before and after CPRT was possible on 18 occasions (in six patients), and the total score of nine clinical symptoms improved from a median (IQR) of 22 (15–32) before CPRT to 6 (2–18) after CPRT. However, the difference was not significant (p=0.288). Significant improvements were observed in the following individual symptoms: dyspnea improved from a median of 4.5 (2.0–6.25) to 0 (0–2) points (p=0.003), depression from 2 (0–5) to 0 (0–3) points (p=0.008), and insomnia from 0 (0–3.25) to 0 (0–2) points (p=0.009), indicating a statistically significant improvement in subjective symptoms potentially attributable to CPRT (Table 3).

In the 11 patients with dyspnea, the NRS improved 15 out of 15 times (100%) before and after CPRT. Among the 15 instances where dyspnea improved, depression also improved in nine cases (60.0%), indicating a relatively frequent coimprovement. Insomnia improved in five cases (33.3%) (Table 4).

The correlation between pleural fluid drainage volume and symptom improvement before and after CPRT (measured as the change in NRS score) showed no correlation with the reduction of dyspnea (r=0.0461). However, although the number of cases was small and the results were not statistically significant, mild positive correlations were observed for general fatigue (r=0.4627, p=0.0532) and nausea (r=0.4657, p=0.0514). Conversely, a significant mild negative correlation was found for constipation, which tended to worsen with larger drainage volumes (r=–0.5352, p=0.0221) (Table 5).

## Discussion

Pleural effusion associated with malignancy (malignant pleural effusion) is caused by direct tumor invasion of the pleura and increased vascular permeability due to malignant pleuritis. It may also result from impaired pleural fluid absorption due to lymphatic obstruction or injury. Additionally, even in patients without cytological or histological evidence of malignancy, pleural effusion may accumulate due to secondary factors such as bronchial stenosis or obstruction caused by the tumor, lymph node involvement, or pulmonary embolism.^2^ Pleural effusions can cause symptoms such as dyspnea, cough, and reduced exercise tolerance, often necessitating hospitalization and prolonged treatment, including oxygen and opioid administration and invasive drainage procedures for symptom relief.^3^ In cases of massive pleural effusions with significant symptoms, definitive treatment with chemotherapy should be considered when antitumor drug efficacy is expected. When control is difficult with diuretics and other medications, drainage, primarily via thoracentesis or pleurodesis, is often effective. Intrapleural catheter placement is used to avoid repeated thoracentesis, and pleurodesis is performed to prevent reaccumulation.^3^ Guidelines in Europe and the United States also indicate that indwelling pleural catheters and pleurodesis are effective in symptom control for malignant pleural effusions.^4,5^

However, CART was developed in Japan^6^ and has been covered by national health insurance since 1981. It is already widely used as a treatment for refractory ascites in patients with liver cirrhosis.^7^ Furthermore, advances in equipment have enabled the complete (undetectable) removal of cellular components, including cancer cells, blood cells, and bacteria,^6^ thereby expanding its indications to malignant ascites in terminal cancer patients. Its safety has also been demonstrated.^8^ In a systematic review by Chen et al. analyzing 2,567 patients (6,013 times) treated with CART for malignant ascites, serum albumin (Alb) levels increased by 0.14 g/dL after CART, and clinical symptoms such as abdominal distension, dyspnea, and general fatigue were markedly improved.^9^ Furthermore, Yamada et al. reported an increase in Alb level of 2.6 mg/dL with CART performed in cancer patients,^10^ and Hanafusa et al. observed significant improvements in performance status, food intake, urine output, body weight, and abdominal girth in a study involving 128 patients (356 times).^11^ Ito et al. also reported significant improvement in the MDASI-J^12^ with symptom scores decreasing from 4.73 to 2.75 and interference scores from 7.05 to 5.12.^13^ Furthermore, in patients with malignant ascites due to advanced gynecological cancers, CART combined with chemotherapy was associated with prolonged overall survival.^14^ The 2021 Guidelines for the Management of Peritoneal Dissemination^15^ also states that although no evidence has been obtained, further demand is expected for chemotherapy in patients with peritoneal metastasis accompanied by massive ascites.^16^

Therefore, the department performs filtration and concentration reinfusion therapy for pleural effusions, and CART for ascites, with the aim of relieving symptoms, improving quality of life, and maintaining nutritional status.

Regarding the usefulness of CPRT, there have been case reports in which refractory pleural effusion following thoracic esophagectomy in a patient with concurrent liver cirrhosis was well controlled,^17^ massive chylothorax after artificial vascular grafting for a distal aortic arch aneurysm was successfully managed,^18^ and refractory pleural effusion due to lung cancer was controlled after placement of an intrathoracic catheter with a subcutaneous port system.^19^ However, a search of PubMed and Ichushi Web (Japana Centra Revuo Medicina) yielded no detailed reports on CPRT.

Therefore, we investigated the effects of CPRT on clinical symptoms and nutritional parameters. In this study, 71 CPRT sessions were performed in 29 patients with massive pleural effusions to reuse albumin in the pleural fluid and improve clinical symptoms. Results showed that serum Alb and TTR values were maintained without significant decrease before and after CPRT. These pre- and post-CPRT results, based on serum Alb and TTR levels, suggest that nutritional status can be maintained even in patients with advanced or recurrent cancer and cachexia. The present findings on nutritional indices following CPRT are consistent with the previously reported results for CART, suggesting that concentrated reinfusion of pleural effusion may be effective not only for symptom relief but also for maintaining nutritional status. Murai et al. reported that interleukin-8 and clinical symptoms are prognostic factors in advanced cancer patients with cachexia. They found that serum Alb and TTR levels were significantly lower in the short-prognosis group compared to the long-prognosis group.^20^ Miura et al. also identified low TTR levels, muscle weakness, and general fatigue as prognostic factors in cancer patients receiving palliative care.^21^ Therefore, the fact that serum Alb and TTR did not significantly decrease even after CPRT in this study may affect prognosis.

Clinical symptoms such as dyspnea, depression, and insomnia improved significantly (Table 3). CPRT was found to improve not only dyspnea but also depressed mood, and insomnia. These symptoms were interrelated, and in some cases, improvement in dyspnea may have contributed to improvements in depression and insomnia. Thus, the results suggest that CPRT contributes not only to symptom improvement but also to enhanced QOL.

Next, we focused on dyspnea and examined its correlation with the volume of pleural fluid drainage. However, no correlation was found between the volume of pleural fluid drainage and the symptomatic relief of dyspnea. The causes of dyspnea in terminal cancer patients include not only pleural effusion but also a variety of other factors, such as cancer-related causes (tumors, pericardial effusion, airway obstruction, carcinomatous lymphangitis, etc.) and causes related to the patient’s overall condition (anemia, ascites, hepatomegaly, respiratory muscle fatigue due to general weakness, fever, anxiety, depression, and psychological stress).^3^ These factors are intricately related, and therefore, the improvement in dyspnea was not proportional to the volume of pleural fluid drainage. Furthermore, dyspnea is considered a subjective symptom, unlike respiratory failure, which is objectively assessed using parameters such as arterial partial pressure of oxygen. However, general fatigue and nausea correlated with the volume of pleural drainage, and symptomatic improvement was achieved. Therefore, in terminal cancer patients requiring symptom control due to pleural effusion, pleural drainage is important. Moreover, it is considered necessary to drain as much pleural fluid as possible while carefully monitoring vital signs. Conversely, a larger volume of pleural fluid drainage may exacerbate constipation, suggesting that attention should be paid to dehydration and that laxative use should be adjusted accordingly.

Complications of CPRT included fever in six cases (8.4%) and pneumothorax requiring degassing in one case (1.4%). The case of pneumothorax occurred in a patient who underwent CPRT seven times, suggesting that caution is warranted when performing CPRT multiple times. Except for this case, the length of hospital stay was not affected. These results suggest that CPRT may be useful in maintaining and improving the QOL for terminal cancer patients with massive pleural effusions.

The current study did not include patients who underwent pleural drainage alone, but only those who underwent CPRT (pleural drainage+filtration-concentration reinjection) to evaluate the usefulness of CPRT. When performing pleural drainage, the Japanese Society for Palliative Medicine’s “Guidelines for Palliation of Dyspnea in Patients with Advanced Disease” suggest that the volume of pleural fluid drainage at one time should be around 1000–1500 mL.^3^ When CPRT was performed, the goal was to drain as much pleural fluid as possible at one time, with many cases involving the drainage of more than 1,000 mL of pleural fluid. By contrast, many patients are in poor condition when only pleural drainage is performed, and at our institution, we limit the volume of pleural fluid drainage to less than 1000 mL at a time. There was a clear difference in the volume of pleural drainage per drainage depending on whether CPRT or only drainage was performed. Due to bias at this point, comparisons were difficult to make, so we reported only the usefulness of CPRT cases.

The limitations of this study were as follows: 1) the criteria for whether to perform CPRT or drainage alone were unclear; 2) the intervals between CPRT, blood tests, and clinical symptom evaluations were not consistent; and 3) it was difficult to evaluate whether the effect on clinical symptoms was due to drainage, reinfusion of the filtered concentrated pleural fluid, or an additive effect. Furthermore, the assessment of nutritional status is influenced by the amount of oral intake and the status of concomitant intravenous nutrition, which is considered an issue for future study.

## Conclusion

CPRT for pleural effusion in terminal patients can be performed safely, is suggested to help maintain nutritional status, and is believed to be useful in improving clinical symptoms.

## Figures and Tables

**Table 1:** Characteristics of patients

Factor	n=29
Sex (Male/Female)	14/15
Age (years, median)	74 (49–89)
Cancer type (Cases)	
Lung	12
bile duct	3
mammary gland	2
colon	2
urinary	2
other	8

**Table 2:** Overview of CPRT

Number of CPRT Median (min–max)	1 (1–14)
Each amount of drained pleural effusion (mL) Median (min–max)	800 (200–2000)
Each amount of reinfused pleural effusion (mL) Median (min–max)	120 (50–330)

CPRT: Cell-free and concentrated Pleural effusion Reinfusion Therapy

**Table 3:** Clinical symptoms before and after CPRT

	before CPRT Median (IQR)	after CPRT Median (IQR)	P value
Pain	0 (0–4)	0 (0–4)	0.274
General fatigue	4 (2–5.25)	0 (0–3.25)	0.533
Anorexia	4.5 (2–6.25)	0 (0–3)	0.110
Dyspnea	4.5 (2–7.25)	0 (0–2)	0.003
Depression	2 (0–5)	0 (0–3)	0.008
Nausea	0 (0–0)	0 (0–0)	0.079
Insomnia	0 (0–3.25)	0 (0–0.5)	0.009
Constipation	0 (0–2)	0 (0–2)	0.971
Dry mouth	2 (0–4.5)	0 (0–3)	0.251
total	22 (14.75–32)	6 (2–17.75)	0.288

CPRT: Cell-free and concentrated Pleural effusion Reinfusion Therapy

**Table 4:** Relationship between dyspnea and other symptoms

Dyspnea n (%)		Depression n (%)	Insomnia n (%)
Improvement 15 (100)	Improvement	9 (60.0)	5 (33.3)
	Unchanged	2 (13.3)	7 (46.7)
	Worsening	4 (26.7)	3 (20.0)

**Table 5:** Correlations between improvement of clinical symptoms and the amount of drained pleural effusion

	before CPRT	after CPRT	effect (difference)	amount of drained pleural effusion	
				r	p value
Pain	0 (0–4)	0 (0–4)	0 (–2–3)	–0.1892	0.4522
General fatigue	4 (2–5.25)	0 (0–3.25)	3 (–0.5–5)	0.4627	0.0532
Anorexia	4.5 (2–6.25)	0 (0–3)	3 (2–5.5)	0.1611	0.5368
Dyspnea	4.5 (2–7.25)	0 (0–2)	4 (2–5)	0.0461	0.8559
Depression	2 (0–5)	0 (0–3)	1 (–0.25–5)	0.2573	0.3026
Nausea	0 (0–0)	0 (0–0)	0 (0–0)	0.4657	0.0514
Insomnia	0 (0–3.25)	0 (0–0.5)	0 (0–2.5)	0.1435	0.5699
Constipation	0 (0–2)	0 (0–2)	0 (–0.5–0.25)	–0.5352	0.0221
Dry mouth	2 (0–4.5)	0 (0–3)	1.5 (–1–2.25)	0.1769	0.4825
total	22 (14.75–32)	6 (2–17.75)	14 (12–16.25)	–0.3149	0.203

data: median (25–75%)

## Supplementary Material

PDF-Japanese

## References

[1] Ohota K. Gan kannwakea ni okeru kyousui fukusui kannri. Tokyo: Shinko Trading; 2010: 87 (in Japanese).

[2] Caraceni A, Cherny N, Fainsinger R, Kaasa S, Poulain P, Radbruch L, De Conno F. Pain measurement tools and methods in clinical research in palliative care: recommendations of an Expert Working Group of the European Association of Palliative Care. J Pain Symptom Manage 2002; 23: 239–255. 11888722 10.1016/s0885-3924(01)00409-2

[3] Japanese Society for Palliative Medicine. Clinical guidelines for the treatment of dyspnea in advanced diseases. Tokyo: Kanehara; 2023: 33–35 (in Japanese).

[4] Feller-Kopman DJ, Reddy CB, DeCamp MM, Diekemper RL, Gould MK, Henry T, Iyer NP, Lee YCG, Lewis SZ, Maskell NA, Rahman NM, Sterman DH, Wahidi MM, Balekian AA. Management of malignant pleural effusions. An official ATS/STS/STR clinical practice guideline. Am J Respir Crit Care Med 2018; 198: 839–849. 30272503 10.1164/rccm.201807-1415ST

[5] Bibby AC, Dorn P, Psallidas I, Porcel JM, Janssen J, Froudarakis M, Subotic D, Astoul P, Licht P, Schmid R, Schrepel A, Rahman NM, Maskell NA, Cardillo G. ERS/EACTS statement on the management of malignant pleural effusions. Eur J Cardiothorac Surg 2019; 55: 116–132. 30060030 10.1093/ejcts/ezy258

[6] Hata Y, Kobayashi R. Characteristics of ascites filters and ascites concentrators. Japanese Journal of Apheresis 2019; 38: 42–47 (in Japanese).

[7] The Japanese Society of Gastroenterology, The Japan Society of Hepatology. Evidence-based clinical practice guidelines for liver cirrhosis. 3rd ed. Tokyo: Nankodo; 2020 (in Japanese).

[8] Tsubokura M, Adegawa Y, Kojima M, et al. Adverse effects of cell-free and concentrated ascites reinfusion therapy for malignant ascites. BMC Cancer 2022; 22: 268. 35287609 10.1186/s12885-022-09298-6 PMC8919605

[9] Chen H, Ishihara M, Horita N, Tanzawa S, Kazahari H, Ochiai R, Sakamoto T, Honda T, Ichikawa Y, Watanabe K, Seki N. Effectiveness of Cell-free and Concentrated Ascites Reinfusion Therapy in The Treatment of Malignancy-Related Ascites: A Systematic Review and Meta-Analysis. Cancers 2021; 13: 4873. 34638357 10.3390/cancers13194873 PMC8508032

[10] Yamada Y, Inui Y, Hara Y, Fuji K, Sonoda K, Hashimoto K, Kamijo Y. Verification of serum albumin elevating effect of cell-free and concentrated ascites reinfusion therapy for ascites patients: a retrospective controlled cohort study. Sci Rep 2019; 9: 10195. 31308465 10.1038/s41598-019-46774-9 PMC6629637

[11] Hanafusa N, Isoai A, Ishihara T, et al. Safety and efficacy of cell-free and concentrated ascites reinfusion therapy (CART) in refractory ascites: Post-marketing surveillance results. PLoS One 2017; 12: e0177303. 28510606 10.1371/journal.pone.0177303 PMC5433707

[12] Okuyama T, Wang XS, Akechi T, Mendoza TR, Hosaka T, Cleeland CS, Uchitomi Y. Japanese version of the MD Anderson symptom inventory: a validation study. J Pain Symptom Manage 2003; 26: 1093–1104. 14654261 10.1016/j.jpainsymman.2003.05.003

[13] Ito T, Hanafusa N, Iwase S, Noiri E, Nangaku M, Nakagawa K, Miyagawa K. Effects of cell-free and concentrated ascites reinfusion therapy (CART) on symptom relief of malignancy-related ascites. Int J Clin Oncol 2015; 20: 623–628. 25239690 10.1007/s10147-014-0750-y

[14] Ueda T, Maehara M, Takahashi Y, Nakayama N, Kondo H, Shirota K, Yoshizato T, Miyamoto S. Clinical significance of cell-free and concentrated ascites re-infusion therapy for advanced and recurrent gynecological cancer. Anticancer Res 2012; 32: 2353–2358. 22641674

[15] Japanese Society of Peritoneal Malignancy. Clinical practice guideline for peritoneal malignancy 2021. Tokyo: Kanehara; 2021 (in Japanese).

[16] Matsusaki K, Aridome K, Emoto S, Kajiyama H, Takagaki N, Takahashi T, Tsubamoto H, Nagao S, Watanabe A, Shimada H, Kitayama J. Clinical practice guideline for the treatment of malignant ascites: section summary in Clinical Practice Guideline for peritoneal dissemination. Int J Clin Oncol 2022; 27: 1–6. 34800177 10.1007/s10147-021-02077-6 PMC8732893

[17] Xiaolin Y, Takeda S, Watanabe Y, Iida M, Yamamoto T, Nakashima C, Nishiyama M, Matsui H, Shindo Y, Tokumitsu Y, Tomochika S, Yoshida S, Suzuki N, Ioka T, Nagano H. Postoperative Management of Refractory Pleural Effusion in a Patient with Esophageal Cancer Accompanied by Cirrhosis. Gan To Kagaku Ryoho 2021; 48: 2036–2038 (in Japanese). 35045485

[18] Takeuchi K, Takahashi N, Hiyama N, Watanabe H. Cell-free and Concentrated Pleural Effusion Reinfusion Therapy Using an lntrathoracic Catheter Subcutaneous Port System Can Achieve a Long-Term Home Care for a Patient with Refractory Pleural Effusion in Lung Cancer. Yamaguchi Medical Journal 2016; 65: 167–172 (in Japanese).

[19] Sawada Y, Nomura Y, Yoshii Y. Cell-Free and Concentrated Pleural Effusion Reinfusion Therapy for Postoperative Chylothorax. Japanese Journal of Cardiovascular Surgery 2009; 38: 205–207 (in Japanese).

[20] Murai M, Higashiguchi T, Futamura A, Ohara H, Tsuzuki N, Itani Y, Kaneko T, Chihara T, Shimpo K, Nakayama N. Interleukin-8 and clinical symptoms can be prognostic indicators for advanced cancer patients with cachexia. Fujita Med J 2020; 6: 117–121. 35111532 10.20407/fmj.2018-022 PMC8761829

[21] Miura T, Amano K, Shirado A, Baba M, Ozawa T, Nakajima N, Suga A, Matsumoto Y, Shimizu M, Shimoyama S, Kuriyama T, Matsuda Y, Iwashita T, Mori I, Kinoshita H. Low transthyretin levels predict poor prognosis in cancer patients in palliative care settings. Nutr Cancer 2018; 70: 1283–1289. 30663397 10.1080/01635581.2018.1557213

