# Supplementary material for: Usefulness of cell-free and concentrated pleural effusion reinfusion therapy in terminal cancer patients
Source: Fujita Med J. 2025 Nov 5;12(1):6–11. doi: 10.20407/fmj.2024-034 (PMC12865283; doi:10.20407/fmj.2024-034)
Supplement: Supplementary file 1 — PDF-Japanese [file fmj-12-006-s001.pdf]

終末期がん患者の胸水貯留に対する胸水濾過濃縮再静注法の有用性
ランニングタイトル　胸水濾過濃縮再静注法の有用性

藤崎宏之[1*]、臼井正信[1*]、伊藤彰博[1]、村井美代[1]、都築則正[1]、二村昭彦[1]、
今井一輝[1]、井谷功典[2]
藤田医科大学医学部　外科・緩和医療学講座[1]
藤田医科大学　七栗記念病院　医療技術部　臨床検査・輸血課[2]

Hiroyuki Fujisaki, MD, PhD[1*], Masanobu Usui, MD, PhD [1*], Akihiro Ito, MD, PhD [1], Miyo Murai, MD, PhD[1], Norimasa Tsuzuki, PhD[1], Akihiko Futamura, PhD[1], Kazuki Imai, PhD[1], Yoshinori Itani[2]
* These two authors contributed equally to this article
[1]Department of Surgery and Palliative Medicine, Fujita Health University, School of
Medicine, Toyoake, Aichi, Japan
[2] Department of Medical Technology, Clinical Examination Division, Fujita Health University Nanakuri Memorial Hospital, Tsu, Mie, Japan

Original Article

Corresponding author: Akihiro Ito, MD, PhD
Department of Surgery and Palliative Medicine, Fujita Health University, School of Medicine, 1-98, Dengakugakubo, Kutsukake-cho, Toyoake, Aichi 470-1192, Japan
Tel: 0562-93-2111
E-mail: itoaki@fujita-hu.ac.jp

【要旨】
目的：進行・再発がん患者はしばしば過剰な体液貯留を来し、その QOL に大きな影響を与える。腹水に対する治療法の一つとして腹水濾過濃縮再静注法が普及しているが、胸水に対する濾過濃縮再静注法（Cell-free and Concentrated Pleural effusion Reinfusion Therapy :CPRT）の詳細な報告はない。そこで、終末期がん患者の胸水貯留に対する CPRT の有用性を検討した。
方法：2016 年 4 月から 2020 年 8 月までに、藤田医科大学七栗記念病院で CPRT を施行した 29 例を対象とし、その効果と合併症を検討した。CPRT 前後の栄養状態は、血清アルブミン（Alb :albumin）値、トランスサイレチン（TTR :transthyretin）

値を指標として用い、臨床症状は、疼痛、倦怠感、食思不振、呼吸困難、嘔気、気分の落ち込み、不眠、便秘、口渇の 9 項目を Numerical Rating Scale にて評価した。
結果：CPRT は延べ 71 回（中央値 1 回）施行し、抜水量の中央値は 800ml（200～2000ml）、還流量は中央値 120ml（50～330ml）であった。CPRT 前後の栄養状態では血清 Alb 値、TTR 値は有意な低下を認めず維持されていた（p=0.568, p=0.944）。臨床症状では 9 項目の加算値は、改善傾向を認めた（p=0.288）。項目別では呼吸困難（p=0.003）、気分の落ち込み（p=0.008）、不眠（p=0.009）が有意に改善していた。
抜水量と呼吸困難の軽減との相関関係は認められなかったが、倦怠感（r= 0.4627)、嘔気（r= 0.4657)は軽度の相関関係を認めた。逆に、便秘は抜水量と軽度の逆相関関係が認められた（r= -0.5352、p＝0.0221)。
結論：終末期がん患者の胸水貯留に対する CPRT は、栄養状態を維持する事が示唆され、臨床症状の改善に有用であると考えられた。

Key words：悪性胸水　胸水濾過濃縮再静注法　CART　栄養状態　臨床症状

【序論】進行・再発がん患者は、病勢の進行、悪液質の合併とともに、全身浮腫、胸水・腹水貯留など、体液の過剰貯留をしばしば認める。胸水の貯留は呼吸困難をきたし、酸素・オピオイドの投与が必要となるなど ADL が低下する要因となる。このため胸水貯留による症状のコントロールは患者の QOL に大きく影響する。治療法としては第一に利尿剤の投与、輸液の減量などが行われるが、胸水が大量に貯留し、特に呼吸困難を呈している場合にはドレナージが行われることが多い。現在本邦においてがん腹膜転移例における大量腹水に対しては、ドレナージだけではなく、腹水濾過濃縮再静注法（Cell-free and concentrated Ascites Reinfusion Therapy; CART）はその治療法として普及している。CART は、腹水濾過器で処理し，細菌やがん細胞，血球成分などを除去し，アルブミンやグロブリン等のタンパク成分を回収し、患者自身に再静注する治療法である。回収蛋白質を再静注することで、血漿膠質浸透圧を保持し、再貯留を抑制、加えて患者の全身・栄養状態の改善による QOL（Quality of Life）の向上を図ると共に、血漿アルブミン製剤による感染性・免疫学的副作用が回避できるという特長がある[1)]。一方で胸水に対しての濾過濃縮再静注法（Cell-free and concentrated Pleural effusion Reinfusion Therapy; CPRT）に関する詳細な報告はほとんどない。そこで今回進行・再発がん患者に対する CPRT の臨床症状や栄養指標に及ぼす効果について報告する。

【方法】2016 年 4 月～2020 年 8 月までの 4 年 5 か月間に、藤田医科大学・七栗記念

病院に緩和医療目的で入院した 1328 例のうち、CPRT を施行した 29 例（2.2%）を対象とした。

本研究は、後ろ向き研究である。当院では入院時すべての患者に対し、入院中に行う検査の値や画像データなどをプライバシーに十分配慮した上で、研究に使用する場合があることを説明し、書面で同意を得ている。今回の研究において同意が得られなかった患者は存在しなかった。CPRT の適応症例に対しては、効果、合併症などについて、患者及び家族へ十分な説明を行い、書面にて同意を得られた後に施行した。CPRT は、胸水貯留に対し、利尿剤の投与、輸液の減量を施行するも効果がなく、特に呼吸困難を訴えている患者を適応としている。但し、CART と同様に血清総ビリルビン値 5mg/dl 以上、38℃以上の発熱・ CRP 高値などより感染が強く疑われる場合、血性胸水の著明な溶血所見がある場合は、CPRT 適応不可とした [1]。さらに、緩和ケア目的の入院であり、胸水穿刺や CPRT を希望しない患者も存在する。このような場合は、患者、家族の希望を最優先し胸水穿刺は施行せず、医療用麻薬などの薬剤投与を駆使し、症状コントロールに努めた。

また本研究は、藤田医科大学医学研究倫理審査委員会（HM16-401）にて承認されている。評価項目として、ドレナージの施行回数、抜水量、合併症の有無および臨床症状の変化について検討した。CPRT 前後の栄養状態の評価として、血液生化学検査を CPRT の施行前 3 週間以内、施行後 3 週間以内で測定していた 11 例について、血清アルブミン（Alb : g/dL）値、トランスサイレチン（TTR : mg/dL）値を CPRT 前後で比較検討した。採血は、朝食前 空腹時に行い、同一血清を用いて分析した。TTR 値は、Espa・TTRⅡreagent を用いて、TIA 法で、Alb 値は、AU reagent ALB を用いて、改良 BCP 法で測定した。これらの血液検体は、AU680 Automatic analyzer（BECKMAN COULTER, Osaka, Japan）を用いて分析した。血清 TTR 濃度の基準値は、22~44mg/dL、Alb 濃度の基準値は、4.0~5.0g/dL であった。

臨床症状は、numerical rating scale[2]を用いて、以下の 9 項目を 0～10 の 11 段階にスコア化し評価した: 痛み、倦怠感、食思不振、不眠、気分の落ち込み、嘔気、不眠、便秘、口渇。さらにこれら 9 項目のスコアを合計し総合的な臨床症状加算式総合評価を作成し、評価した(Figure 1) 。入院時と、以降は週に 1 回聞き取りを行い評価している。

胸水ドレナージは、局所麻酔下にアスピレーションキット（COVIDIEN 社、Argyle 8Fr）を用い施行した。回収液は専用バッグ（旭化成メディカル社）に回収した。回収液は、腹水ろ過器 AHF-MO、腹水濃縮器 AHF-UF（旭化成メディカル社）を使用し濾過濃縮処理を行い、約 100ml/hr にて再静注した。すなわち、まず濾過膜で原胸水からがん細胞、血球、細菌、フィブリンなどを分離除去した後に、濃縮膜で余分な水分、電解質を除去して最終的に総量が約 1/10 前後のアルブミン・グロブリン濃縮液を作成し、再静注した。一度に大量の胸水排液を短時間に行うと、再膨張性肺水腫を生じる可能性があるため、日本緩和医療学会“進行性疾患患者の呼吸困難の緩和に

関する診療ガイドライン”の提唱に従い、1 回の排液を 1000-1500ml 程度とした [3)]。原則として発熱予防等の理由でのステロイドの前投薬は施行していない。

統計学的検定

連続変数は、Mann–Whitney U test を、カテゴリー変数は、Fisher's exact test を用いて分析を用い、P<0.05 を有意差ありと判定した。また、相関関係は Spearman の相関係数（r）を求め、P<0.05 を有意差あり、0.4≦r≦1.0 を正の相関関係あり、-1.0≦r≦-0.4 を負の相関関係ありと判定した。

【結果】

背景因子として、年齢の中央値は 74 歳（49～89 歳）、男女比は 14:15 であった。原発巣は、肺がん 12 例（40.0%）、胆管、乳がん各 3 例（10.0%）と多く、大腸、腎・尿管がん（各 2 例）、胃、膵、腹膜、子宮、胸腺、下顎がん、胸腔内骨肉腫（各 1 例）であった。がん性胸膜炎を含む転移性肺がんを伴っていたものは 8 例であった（Table 1）。

CPRT の施行回数は延べ 71 回で、中央値は 1 回(範囲：1～14 回)と半数以上の症例は 1 回のみの施行であり、最大 14 回施行していた。抜水量中央値は 800ml（200～2000ml）、還流量は中央値 120ml（50～330ml）であった（Table 2）。合併症は、38℃以上の発熱を 6 例（8.4%）に、脱気が必要となった気胸を 1 例（1.4%）に認めた。

CPRT 前後の栄養状態の推移として評価できたのは、血液生化学検査を CPRT の施行前 3 週間以内、施行後 3 週間以内で測定していた 11 例であった。血清 Alb 値は、CPRT 前の中央値（四分位範囲）で 2.3（1.9～3.7）g/dl に比べ CPRT 後は中央値 2.4（1.4～3.7）g/dl と有意差無く保たれていた（p=0.568)。また血清 TTR 値は CPRT 前の中央値 13.3（9.3～20.8）g/dl に比べ CPRT 後は中央値 10.2（7.6～20.7）g/dl と有意差は無く保たれていた（p=0.944）（Fig.2）。

CPRT 前後の臨床症状を加算式評価で可能であったのは 18 回（6 例）で、臨床症状 9 項目の合計点の推移は、CPRT 前の中央値（四分位範囲）で 22 (15～32)点から CPRT 後 6(2～18) 点に改善していたが、有意差は認めなかった（p=0.288)。各項目は、それぞれ中央値（四分位範囲）で、呼吸困難 4.5（2.0~6.250）点から 0（0~2）点（p=0.003）、気分の落ち込み 2（0~5）点から 0（0~3）点（p=0.008）、不眠 0（0~3.250）点から 0（0~2）点（p=0.009）と有意に改善しており CPRT の効果と考えられる自覚症状の改善が認められた（Table 3）。

呼吸困難のあった患者 11 名では、CPRT 前後で NRS は 15 回中 15 回（100%）で改善を認めた。この呼吸困難が改善した 15 回の中で、気分の落ち込みは、9 回（60.0%）と比較的高率に改善していた。不眠の改善は 5 回（33.3%）であった。(Table 4)。

抜水量と CPRT 前後の症状効果（CPRT 前の NRS 値－CPRT 後の NRS 値）の相関関係では、抜水量と呼吸困難の軽減との間に相関関係は認められなかった(r= 0.0461)。しかし症例数が少なく有意差は認めなかったが、倦怠感（r= 0.4627、p＝0.0532)、嘔気（r= 0.4657、p＝0.0514)は軽度の相関関係を認めた。逆に、便秘は抜水量が多くなると悪化する、有意の負の軽度相関関係が認められた（r= -0.5352、p＝0.0221）（Table 5)。

【考察】

悪性腫瘍に伴って生じる胸水(悪性胸水)は、腫瘍が胸膜に直接浸潤し、がん性胸膜炎により血管の透過性が亢進し、引き起こされる。また、リンパ管の障害や損傷による胸水の吸収障害によっても引き起こされることがある。さらに胸水細胞診や胸膜生検などで悪性所見を証明できない症例においても、腫瘍が原因で生じる気管支狭窄・閉塞、リンパ節浸潤、肺塞栓など二次的要因により胸水が貯留することもある [2)]。胸水貯留は呼吸困難、咳嗽、運動耐容能の低下などの症状を来し、症状緩和のため酸素やオピオイドの投与、侵襲的ドレナージ処置が必要となる [3)]など、入院治療、長期間の治療を余儀なくされる場合があり、予後が限られた終末期がん患者にとって QOL を大きく損なうことになる。臨床症状を伴う大量胸水に対する治療戦略は、抗腫瘍薬の治療効果が期待できる場合には、化学療法による根本治療を考慮する。利尿薬などの薬物療法でコントロールが困難な場合、ドレナージにより改善することが多く、主に胸水穿刺ドレナージが行われる。頻回穿刺を避けるために胸腔内カテーテル留置、再貯留予防のためには胸膜癒着が行われる [3)]。欧米でのガイドラインにおいても症候性悪性胸水に対して、胸膜留置カテーテル、胸膜癒着術が症状コントロールに有効であるとされている [4)5)]。

一方、CART は本邦で開発され [6)]、1981 年に保険収載された治療法で、すでに肝硬変の難治性腹水に対する治療として広く普及している [7)]。さらに装置の進歩によりがん細胞や血球、細菌を含む細胞成分の 100%除去(未検出)が可能となった事で [6)]、終末期がん患者のがん性腹水に対して適応拡大された。またその安全性も検証されている [8)]。Chen らの、Systematic review にて 2567 例（6013 回）の悪性腹水に対する CART の解析では、CART 後に Alb 値は 0.14mg/dL 上昇し、臨床症状として腹部膨満感、呼吸困難、倦怠感が著明に改善されたと報告されている [9)]。　さらに、Yamada らはがん患者に施行した CART により Alb 値が 2.6mg/dL 上昇したと報告し [10)]、Hanafusa らは CART を施行した 128 例(356 回)の検討において、PS、食事量、尿量、体重、腹囲が有意に改善することを報告している [11)]。また Ito らは MDASI-J[12)] においても症状スコアが 4.73 から 2.75、生活支障スコアは 7.05 から 5.12 に有意に改善を認めたと報告している [13)]。更に進行性婦人科がんの悪性腹水に対しては、化学療法との併用で全生存率を延長したと報告されている [14)]。腹膜播種診療ガイドライン(2021 年版) [15)]においても、エビデンスは得られていないものの、

今後は大量の腹水を伴う腹膜播種転移症例の化学療法において一層の需要が見込まれるとしている[16)]。

そこで当科では胸水に対しても症状緩和、QOL の改善だけでなく栄養状態の保持を目的として、腹水に対する CART と同様、濾過濃縮再静注法を行っている。

CPRT の有用性に関しては、肝硬変併存胸部食道癌切除例における難治性胸水に対してコントロール良好となった例[17)]や、遠位弓部大動脈瘤に対する人工血管置換術後の大量乳び胸に対してコントロール良好となった例[18)]、肺がんによる難治性胸水に対して胸腔内カテーテル皮下ポートシステムを留置しコントロール良好となった例[19)]などの症例が報告されている。しかし CPRT についての詳細な報告は PubMed、医学中央雑誌での検索では見当たらなかった。

このため、今回 CPRT の臨床症状と栄養指標に及ぼす効果に関して検討を行った。

本研究では 29 例の大量胸水貯留患者に対し、胸水中に含まれる Alb の再利用や臨床症状の改善を目的に 71 回の CPRT を施行した。その結果、Alb 値と TTR 値は CPRT 前後で有意な低下は認めず、維持された。この血清 Alb 値、TTR 値による CPRT 前後の結果は、悪液質状態の進行・再発がん患者においても、栄養状態は維持されることが示唆された。今回の CPRT における栄養指標に関する結果は前述の CART の結果に近く、胸水の濃縮再静注も、症状緩和だけでなく栄養状態の維持という目的においても有効である可能性が示唆された。Murai らは、悪液質を伴う進行がん患者に対して、Interleukin-8 や臨床症状が予後規定因子であり、予後が短い群は、長い群に比較して、血清 Alb、TTR 値は、有意に低値であると報告している[20)]。また Miura らは緩和ケアを受けているがん患者では低 TTR 値、筋力低下、倦怠感が予後因子となるとしている[21)]。したがって本研究で血清 Alb、TTR が CPRT 後でも有意な低下を認めなかったことから、予後に影響する可能性が考えられた。

臨床症状では呼吸困難、気分の落ち込み、不眠などが有意に改善していた（Table 3）。CPRT は、呼吸困難が改善することはもちろんのこと、気分の落ち込み、不眠にも効果が認められた。これらの症状は、それぞれ関連しており、一部の症例では、呼吸困難の改善が、気分の落ち込みや不眠の改善に影響した可能性が考えられた。

このように CPRT は症状改善のみならず QOL の向上にも寄与していることが示唆された。

次に、呼吸困難に注目し、抜水量との相関関係を検討した。しかし抜水量と呼吸困難の症状緩和に関しては、相関関係は認めなかった。終末期がん患者における呼吸困難の原因としては、胸水貯留だけではなく、がんに関連した原因（腫瘍、心嚢水、気道閉塞、がん性リンパ管炎など）や全身状態が原因（貧血、腹水、肝腫大、全身衰弱に伴う呼吸筋疲労、発熱、不安、抑うつ、精神的ストレス）[3)]など様々な要因があり、これらが複雑に関連しているため、抜水量に比例して、呼吸困難改善をもたらすという結果に繋がらなかったと考えられた。さらに、呼吸困難は、動脈血酸素分圧などの客観的評価である呼吸不全とは異なり、主観的評価であるための結

果であることも一因ではないかと考えられた。しかし、倦怠感や嘔気は抜水量に相関して、症状改善が得られていた。したがって、胸水貯留にて症状コントロールが必要な終末期がん患者に対しては、胸水抜水を施行することが重要であり、加えてVital sign を十分に考慮しながら、少しでも多くの胸水をドレナージすることも必要であると考えられた。逆に、便秘は抜水量に応じて悪化する可能性があり、脱水に注意し、便秘薬の調整が必要であると考えられた。

CPRT の合併症は発熱を 6 例（8.4%）、脱気処置を行った気胸を 1 例（1.4%）に認めた。気胸の 1 例は CPRT を 7 回行った症例であり、複数回施行する際には注意が必要である。この症例以外は入院期間に影響はなかった。これらの結果は、終末期がん患者で大量胸水が貯留している際に、CPRT が QOL の維持、向上に有用であることを示唆していると考えられた。

今回の研究は、胸水抜水（ドレナージ）のみを施行した症例は対象とせず、CPRT（胸水抜水＋濾過濃縮再静注）を施行した症例のみの有用性の検討を行った。胸水抜水を施行する場合、日本緩和医療学会“進行性疾患患者の呼吸困難の緩和に関する診療ガイドライン”の提唱に従い、1 回の胸水抜水量を 1000-1500ml 程度としている [3)]。CPRT を施行する場合は、1 回の胸水抜水量をできる限り多くするように計画し、多くの症例が 1000ml 以上の抜水を実行していた。それに比し、胸水抜水のみの場合、状態が悪い人も多く当科では 1 回の抜水量を 1000ml 以下にしている。CPRT を施行するか、抜水（ドレナージ）のみを施行するかにより、1 回の抜水量に明らかに差があり、この時点でバイアスがかかっているため、比較は難しく、CPRT 症例のみの有用性を報告した。
本研究の限界としては、①CPRT を施行するか、ドレナージのみとするかの適応基準が不明確であったこと、②血液検査施行時期、臨床症状の評価時期の CPRT との間隔が一定しないこと、③臨床症状に対する効果がドレナージによるものか、濾過濃縮液の再静注によるものか、上乗せ効果なのか評価が困難なことが問題点と考えられた。更に栄養状態の評価は、経口摂取量や静脈栄養併用の状況に影響されるため、今後の検討課題と考えられた。

【結論】終末期がん患者における胸水貯留に対する胸水濾過濃縮再静注法（CPRT）は、安全に施行でき、栄養状態の維持が示唆され、臨床症状の改善に有用であると考えられた。

利益相反著者は、本研究に関わる企業や営利目的とした団体との利益相反はありません。

【参考文献】

1) Ohota K. Gan kannwakea ni okeru kyousui fukusui kannri. Tokyo: Shinko Trading; 2010: 87(in Japanese).
2) Caraceni A, Cherny N, Fainsinger R, Kaasa S, Poulain P, Radbruch L, De Conno F. Pain measurement tools and methods in clinical research in palliative care: recommendations of an Expert Working Group of the European Association of Palliative Care. J Pain Symptom Manage 2002; 23: 239-55.
3) Japanese Society for Palliative Medicine. Clinical guidelines for the treatment of dyspnea in advanced diseases. Tokyo: Kanehara;2023 : 33-5(in Japanese).
4) Feller-Kopman DJ, Reddy CB, DeCamp MM, Diekemper RL, Gould MK, Henry T, Iyer NP, Lee YCG, Lewis SZ, Maskell NA, Rahman NM, Sterman DH, Wahidi MM, Balekian AA. Management of malignant pleural effusions. An official ATS/STS/STR clinical practice guideline. Am J Respir Crit Care Med 2018; 198: 839-49.
5) Bibby AC, Dorn P, Psallidas I, Porcel JM, Janssen J, Froudarakis M, Subotic D, Astoul P, Licht P, Schmid R, Schrepel A, Rahman NM, Maskell NA, Cardillo G. ERS/EACTS statement on the management of malignant pleural effusions. Eur J Cardiothorac Surg 2019; 55: 116-32.
6) Hata Y, Kobayashi R. Characteristics of ascites filters and ascites concentrators. Japanese Journal of Apheresis 2019; 38: 42-7(in Japanese).
7) The Japanese Society of Gastroenterology, The Japan Society of Hepatology. Evidence-based clinical practice guidelines for liver cirrhosis. 3rd ed. Tokyo: Nankodo; 2020(in Japanese).
8) Tsubokura M, Adegawa Y, Kojima M, et al. Adverse effects of cell-free and concentrated ascites reinfusion therapy for malignant ascites. BMC Cancer 2022; 22:268.
9) Chen H, Ishihara M, Horita N, Tanzawa S, Kazahari H, Ochiai R, Sakamoto T, Honda T, Ichikawa Y, Watanabe K, Seki N. Effectiveness of Cell-free and Concentrated Ascites Reinfusion Therapy in The Treatment of Malignancy-Related Ascites: A Systematic Review and Meta-Analysis. Cancers 2021; 13: 4873.
10) Yamada Y, Inui Y, Hara Y, Fuji K, Sonoda K, Hashimoto K, Kamijo Y. Verification of serum albumin elevating effect of cell-free and concentrated ascites reinfusion therapy for ascites patients: a retrospective controlled cohort study. Sci Rep 2019; 9:10195.
11) Hanafusa N, Isoai A, Ishihara T, et al. Safety and efficacy of cell-free and

concentrated ascites reinfusion therapy (CART) in refractory ascites: Post-marketing surveillance results. PLoS One 2017;12: e0177303.
12) Okuyama T, Wang XS, Akechi T, Mendoza TR, Hosaka T, Cleeland CS, Uchitomi Y. Japanese version of the MD Anderson symptom inventory: a validation study. J Pain Symptom Manage 2003; 26: 1093-104.
13) Ito T, Hanafusa N, Iwase S, Noiri E, Nangaku M, Nakagawa K, Miyagawa K. Effects of cell-free and concentrated ascites reinfusion therapy (CART) on symptom relief of malignancy-related ascites. Int J Clin Oncol 2015; 20:623-8.
14) Ueda T, Maehara M, Takahashi Y, Nakayama N, Kondo H, Shirota K, Yoshizato T, Miyamoto S. Clinical significance of cell-free and concentrated ascites re-infusion therapy for advanced and recurrent gynecological cancer. Anticancer Res 2012; 32: 2353-8.
15) Japanese Society of Peritoneal Malignancy. Clinical practice guideline for peritoneal malignancy 2021. Tokyo: Kanehara;2021 (in Japanese).
16) Matsusaki K, Aridome K, Emoto S, Kajiyama H, Takagaki N, Takahashi T, Tsubamoto H, Nagao S, Watanabe A, Shimada H, Kitayama J. Clinical practice guideline for the treatment of malignant ascites: section summary in Clinical Practice Guideline for peritoneal dissemination. Int J Clin Oncol 2022; 27: 1-6.
17) Xiaolin Y, Shigeru T, Yusaku W. Postoperative Management of Refractory Pleural Effusion in a Patient with Esophageal Cancer Accompanied by Cirrhosis. Jpn J Cancer Chemother. 2021; 48: 2036-38 (in Japanese)
18) Kana T, Naoko T, Noriko H, Hiroyuki W. Cell-free and Concentrated Pleural Effusion Reinfusion Therapy Using an lntrathoracic Catheter Subcutaneous Port System Can Achieve a Long-Term Home Care for a Patient with Refractory Pleural Effusion in Lung Cancer. Yamaguchi Igaku. 2016; 65: 167-72. (in Japanese)
19) Yoshihide S, Yukiya N, Yasuyoshi Y. Cell-free and Concentrated Pleural Effusion Reinfusion Therapy for Postoperative Chylothorax. Jpn J Cardiovasc. Surg. 2009; 38: 205-7 (in Japanese)
20) Murai M, Higashiguchi T, Futamura A, Ohara H, Tsuzuki N, Itani Y, Kaneko T, Chihara T, Shimpo K, Nakayama N. Interleukin-8 and clinical symptoms can be prognostic indicators for advanced cancer patients with cachexia. Fujita Med J 2020; 6: 117-21.
21) Miura T, Amano K, Shirado A, Baba M, Ozawa T, Nakajima N, Suga A, Matsumoto Y, Shimizu M, Shimoyama S, Kuriyama T, Matsuda Y, Iwashita T, Mori I, Kinoshita H. Low transthyretin levels predict poor prognosis in

cancer patients in palliative care settings. Nutr Cancer 2018; 70: 1283-9.

legend for figures

Figure 1 Overall assessment score
Table 1 Characteristics of patients
Table 2 Overview of Cell-free and concentrated Pleural effusion Reinfusion Therapy (CPRT)
Table 3 Clinical symptoms before and after CPRT
Figure 2 Changes in serum albumin and transthyretin levels before and after CPRT
Table 4 Correlations between improvement of clinical symptoms and the amount of drained pleural effusion
Table 5 Relationship between dyspnea and other symptoms
